# Prevention of Dementia: Focus on Lifestyle

**DOI:** 10.4061/2010/393579

**Published:** 2010-06-29

**Authors:** Maria Cristina Polidori, Gereon Nelles, Ludger Pientka

**Affiliations:** ^1^Department of Geriatrics, Marienhospital Herne, Ruhr University Bochum, 44627 Herne, Germany; ^2^Department of Neurology, St. Elisabeth-Krankenhaus Köln, 50935 Cologne, Germany

## Abstract

The objective of this paper is to summarize current knowledge on the possible advantages of lifestyle interventions, with particular attention to physical fitness, cognitive activity, leisure and social activity as well as nutrition. There is a large amount of published papers providing partial evidence and asserting the need for immediate, appropriate preventive lifestyle measures against dementia and AD development. Nevertheless, there are currently great difficulties in drafting effective guidelines in this field. This depends mainly upon lack of randomized controlled trials assessing benefits versus risks of particular lifestyle interventions strategies. However, due to the rapid increase of dementia burden, lifestyle factors and their amelioration should be already made part of decision making in light of their health-maintaining effects while awaiting for results of well-designed large prospective cohort studies in dementia.

## 1. A Brief Update on the Epidemiology of Dementia

In developed countries, the percentage of subjects aged 60 years and older will increase from 19% currently to almost 40% in year 2050 [1]. About three-quarters of the 1.2 billion over-60 year-old subjects, however, will reside in the least developed and developing countries by 2025 [2]. Important with this trend is that the older population itself ages, as the group of very old people (aged 80 years and older) is projected to grow as much as eight to ten times on the global scale by 2050 [3]. These projections lead to the alarming figure of a steady, apparently unavoidable increase of certain age-related diseases including neurodegenerative diseases in general and dementia in particular. General practitioners, geriatricians, neurologists, and health care professionals all over the world will be facing by 2040 the diagnostic, therapeutic and socioeconomical challenges of over 80 million people with dementia, 70% of which will be residing in the least developed world countries. There are currently 18 million people with dementia in Europe, Africa, Asia and Latin America, and nearly 29 million demented subject are predicted by 2020 [2]. In 2007, more than 5 million people in the US suffered from Alzheimer's disease (AD). If on one hand these numbers are impressive enough, there is a striking possibility of underestimation, not only in developing countries, due to factors likely prevalent among dementia patients and their relatives, such as inadequate diagnosis, lack of awareness, and low education [4]. 

Dementia is one of the most common diseases in the elderly, with crude prevalence rates between 5.9%–9.4% for subjects aged over 65 in the European Union [5]. The lowest age- and gender-specific prevalence of all-causes dementia reported in the literature is 61.1% among women aged 100 or greater [6], so that the question “if we live long enough, will we all be demented?” is becoming a gravely recurrent one [7, 8]. Dementia drastically affects daily life and everyday personal activities, is often associated with behavioural symptoms, personality change, and numerous clinical complications, it increases the risk for urinary incontinence, hip fracture, and—most markedly—dependence on nursing care. Thus, it is not surprising that the costs of care for patients with dementia are immense [9].

## 2. The Meaning of Prevention in Dementia

Prevention is key of every public health-related policy. The impressive growth of dementia in terms of incidence and prevalence occurred in the past recent years and its prospected epidemic mark for the immediate future are not the only features characterizing this disease. The other imposing trait of dementia is the lack of effective curative and prevention strategies, made worse by the uncertain diagnosis and the insufficient standardization of screening tools. While current therapeutic aspects of dementia were recently extensively discussed elsewhere [10], the identification of a multimodal approach might help to balancing and complementing the massive pharmacological efforts (mainly focused on directly influencing the amyloidogenic pathway in the brain) usually considered as the principle antidementia, cognition-maintaining strategy, which however are associated with several major pitfalls.

**Figure 1:**
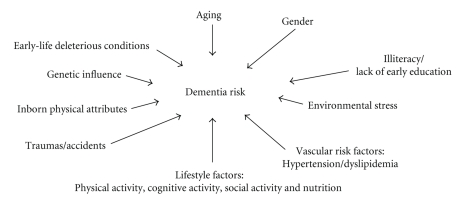
Network of factors possibly and/or certainly influencing dementia risk. Factors specifically addressed in this paper are highlighted in bold.

This paper will focus on specific aspects of dementia prevention. Prevention appears to be particularly prominent among antidementia strategies not only—negatively seen—due to the lack of cure for dementia, but mainly—constructively approached—because it can be carried out within a multidimensional scheme with the highest chances of success if adopted in the early adulthood. Primary prevention is directed against dementia prior to its biological onset or against dementia's risk factors, while secondary prevention refers to the early detection of asymptomatic disease, although the US Preventive Services Task Force suggests there is insufficient evidence to support instituting a universal dementia screening [11]. Syndromes of cognitive impairment in nondemented older adults have been the focus of studies aiming to identify subjects at high risk to develop dementia. Mild cognitive impairment (MCI) is characterized by isolated memory deficits in nondemented persons with subjective memory problems, normal general cognitive functioning, and intact activities of daily living [12]. In the attempt of avoiding dementia development, there are several risk factors to be taken into account (Figure 1), some of which are nonmodifiable and include age with age-influencing early-life deleterious conditions [13], gender, and genetic influence [14]. In addition, there are several inborn physical attributes, factors such as illiteracy and lack of early education, environmental stress, as well as fortuitous circumstances including accidents and traumas that have been associated with increased risk for dementia [13, 15]. Finally, a great deal of attention is being dedicated to the identification and modulation of those factors which have a large potential to be modified before the onset (primary prevention) or during the course (secondary prevention) of the disease. These include vascular and lifestyle factors (Figure 1). Among vascular risk factors, considerable evidence from randomized controlled trials and longitudinal cohort studies has established the relationship between hypertension and dementia as well as between hyperlipidemia and dementia. Both systolic hypertension above 160 mmHg and serum cholesterol above 6.5 mmol/L are known to be associated with an increased RR of 1.5 and 2.1 to develop AD (reviewed in [15]). Based on the recommendations of the Third Canadian Consensus Conference on Diagnosis and Treatment of Dementia held in March 2006, treating systolic hypertension in over 60-year-old subjects to achieve a value of 140 mmHg and below will reduce the risk of dementia with a first level of evidence [15]. By contrast, the same Consensus agreed that acetylsalicylic acid, statin therapy and carotid artery stenosis reopening on a first level of evidence as well as control of type 2 diabetes mellitus, hyperlipidemia and hyperhomocysteinemia on a second level of evidence should not be recommended with the single specific purpose of reducing the risk of dementia [15].

To avoid redundancies, and in light of the large amount of review articles recently published on nonmodifiable and vascular risk factors for dementia as well as on low-evidence preventive effects of medications such as nonsteroidal anti-inflammatory drugs, estrogen and vitamin supplements, and single micro/macronutrients [9, 13–15], we chose to dedicate the following paragraphs to those measures generally referred to as lifestyle factors. Smoking and drinking habits are purposefully left out for the certain negative effects of the former and only possibly positive effects of the latter, if consumed in moderate quantities [16–18]. In contrast to the large amount of published overview and original articles providing partial evidence and asserting the need for immediate, appropriate preventive lifestyle measures against dementia and AD development, however, there are currently great difficulties in drafting effective guidelines in this field [19]. This depends mainly upon lack of randomized controlled trials assessing benefits versus risks of particular lifestyle interventions. In the phenomenologic continuum of later life cognitive decline leading to the very high prevalence of dementia, cognitive reserve plays an inescapable role. Evolution has shaped the human brain through the use of genes, but learning, environment and lifestyle allow the necessary reserve for the brain's *multiple, complex, and redundant *roles and functions. Nutritional, behavioural, physical, and cognitive rules are likely to be most effective to delay the onset of a disease whose various severity grades typically constitute a continuum and often take decades to translate from normal brain aging, to subjective cognitive impairment, to mild cognitive impairment and finally to dementia in its mild, moderate or severe forms.

The objective of this paper is therefore to summarize current knowledge on the possible advantages of lifestyle interventions, with particular attention to physical fitness, cognitive activity, leisure and social activity as well as nutrition. Literature retrieval was accessed through PubMed using the keywords physical activity/exercise, leisure/cognitive/social activity, as well as nutrition/diet and dementia. Major studies published after 1990 were reviewed if they included changes in cognition and a late diagnosis of dementia and AD as an outcome and addressed physical activity, cognitive activity, social activity, and eating behaviors.

## 3. Lifestyle-Related Risk Factors for Dementia and Possible Effects of their Modification

### 3.1. Physical Activity

Physical activity has been suggested to attenuate the pathophysiology of dementia. “Physical activity” refers to “usual care plus physical activity.” Patients and families often ask the physician whether exercise will improve their memory or prevent dementia. Regular physical exercise is an important element in overall health promotion and studies conducted since early 90's showed that it might be an effective strategy to delay the onset of dementia through sustained cerebral perfusion [20]. More recently, Colcombe and Kramer [21] showed that reduced loss of hippocampal brain tissue in the aging brain is related to the level of physical fitness, in agreement with animal studies also showing increased brain cortical thickness with voluntary exercise [22] and other positive brain changes ultimately leading to a preventive effect, with physical activity, on inflammatory pathways and disturbed growth factor signalling [23]. The encouraging results of these studies prompted the performance of longitudinal and randomized trials, which overall confirm that physical exercise enhances cognitive function in older adults [24–33] (Table 1). The association between physical and cognitive function in elderly persons found in several studies has been limited by their cross-sectional design and by the frequent lack of adjustment for potential confounding variables. In a prospective study of 5925 women aged 65 years or older without baseline cognitive impairment or physical limitations, cognitive performance measured by a modified Mini-Mental State Examination (MMSE) at baseline and 6 to 8 years later was shown to remain substantially stable at follow-up in those women performing the highest degree of physical activity (walked blocks, climbed stairs and expended total kilocalories) [34]. Cognitive decline occurred indeed in 17%, 18%, 22%, and 24% of those in the highest, third, second, and lowest quartile of blocks walked per week. After adjustment for age, educational level, comorbid conditions, smoking status, estrogen use, and functional limitation, women in the highest quartile remained less likely than women in the lowest quartile to develop cognitive decline [34]. However, the self-reported nature of the performed physical exercise and the low specificity of the cognitive tests used in several studies still potentially impair the reproducibility of the results. This might explain the lack of benefit of physical exercise in preserving cognitive function observed elsewhere [35]. Even in this negative study, however, dancing was found to be associated with a lower risk of dementia [35]. Larson and colleagues, who reported that regular exercise is associated with a delay in onset of dementia and AD in a population of 1740 persons aged 65 years followed up for over 6 years [31], saw the main limitation of their study in the self-reported way to address exercise frequency by study participants.

An objective measure of movement such as actigraphy, which involves wearing a watch-like device that objectively quantifies accelerometer motion, might be therefore more appropriate to assess the influence of physical training on cognitive measures. A recent study conducted on 2736 older women without evidence of dementia undergoing the assessment of daytime movement over 3 days as assessed by actigraphy, women in the highest movement quartiles had significantly better mean cognitive test scores than those in the lowest quartile and were less likely to be cognitively impaired (odds ratio (OR) = 0.61, 95% confidence interval (CI) = 0.41–0.92 for Trails B; OR = 0.68, 95% CI = 0.44–1.07 for MMSE) [48]. When 134 nursing home residents with AD where divided in two groups undergoing either usual care or collective exercise for 60 minutes twice a week, a slower disability decline and increased gait speed were shown after 12 months of intervention in the group undergoing collective exercise compared to the usual care group [49].

In summary, studies produced so far in the field of physical activity and dementia prevention (either primary or secondary) in the elderly are not comparable and guidelines for the primary and secondary prevention of dementia cannot be drafted. This is due to the self-addressed nature of cognitive performance and performed physical activity as well as to the populations studied (mainly nursing home residents [49], compliance, to the time-window of applied physical intervention, and, in general, to the dyshomogeneity of the methods applied. Physical activity training ranges from 150 min five times per week to 20 min three times per week). Occupational therapies sometimes included in studies on physical activity and dementia prevention also confound results on the latter issue, as no or insufficient evidence is present for the efficacy of counselling the primary caregiver of dementia patients about maintaining the patient's cognitive or functional abilities, respectively [50]. The possibility that physical activity may substantially enhance the brain reserve [51] of the individual is also a critical one which needs to be carefully explored. However, future research in the efficacy of occupational therapy in elderly patient with dementia is recommended. The type of exercise used in future studies should be carefully taken into account, as three recent randomized exercise trials (reviewed in [51]) involving resistance training among seniors provide evidence that also resistance training, in addition to aerobic training, may have cognitive benefits. This might be possible via mechanisms involving IGF-1 and homocysteine in addition to those related to control of dyslipidemia, body mass index, and weight loss [52, 53].

No randomized controlled trials are available which demonstrate that regular physical activity prevents dementia in patients with mild cognitive impairment. Only one RCT on 138 adults aged 50 years and older with subjective memory impairment showed that a 6-month program of physical activity provided a modest improvement in cognition over an 18-month follow-up period [27]. The issues of optimal strategy in terms of intensity, type and duration of the exercise remain matter of additional research. As most recently reviewed in the Cochrane Database System, the results from one meta-analysis and two randomized controlled trials suggest that there is insufficient evidence of the effectiveness of physical activity programs in managing or improving cognition, function, behaviour, depression, and mortality in people with dementia [54]. In addition, family caregiver outcomes and use of health care services are not reported in most of the studies published so far.

If on one hand there is insufficient evidence enabling to say whether or not physical activity programs are beneficial for people with dementia, there is a wealth of relevant articles on the best-designed and performed recent studies confirming longitudinal and short-term RCT evidence that physical activity improves cognitive function in older subjects. Therefore, the current recommendations of regular physical activity as a key component of successful aging can be given to both healthy adults and to elderly subjects with and without cognitive impairment. While awaiting for the results of large ongoing randomized controlled trials in the coming decade, it is to recommend that persons with MCI try to pursue a moderate but regular, variable exercise program consisting of at least 30 minutes three times weekly of walking alternating with aerobically challenging exercise and group sports.

### 3.2. Cognitive Activities, Leisure Activities, and Socialization

The concept of cognitive reserve suggests that innate intelligence or aspects of life experience like educational or occupational attainments may supply reserve in the form of a set of skills or repertoires that allows some people to cope with progressing AD pathology better than others [51]. There is epidemiological evidence that lifestyle characterized by engagement in leisure activities of intellectual and social nature is associated with slower cognitive decline in healthy elderly and may reduce the risk of incident dementia. There is also evidence from functional imaging studies that subjects engaging in such leisure activities can clinically tolerate more AD pathology. It is possible that aspects of life experience like engagement in leisure activities may result in functionally more efficient cognitive networks and therefore provide a cognitive reserve that delays the onset of clinical manifestations of dementia.

Intellectually challenging activity of various types has been associated with a reduced risk of dementia in longitudinal studies [29, 36, 38–42, 54] (Table 1), but there are currently no published randomized controlled trials to provide first level of evidence on this. Daily mental activities have been associated with a decreased risk (RR 0.59) of all-cause dementia in the Kungsholmen Study [37, 42], while the Bronx Aging Study [35] demonstrated that a high level of participation in cognitive leisure activities is associated with a decreased risk of amnestic MCI in community dwellers. In the Washington Heights Study [36], participants who engaged in a higher level of leisure activities (self-reported participation in over 6 among 13 activities versus a low activity level) in the previous month were less likely to develop all-cause dementia. A recently published follow-up study of a RCT of cognitive training appeared to show sustained improvement in specific cognitive performance up to 5 years after the intervention [39]. Wilson et al. [41] recently showed that continuous cognitive activation is associated with reduced evidence of MCI development and that the level of cognitively stimulating activity in old age is related to the risk of developing dementia. However, as in the previous studies, the assessment and collection data of cognitive activity participation was performed through a pooled analysis of past and current frequency of participation in several activities based upon a structured questionnaire containing tens of items. The additional problem in the interpretation of the results complicating the frequent inclusion of physical activity among leisure activities is that the latter include extremely different stimuli going from reading books or newspapers, to writing for pleasure, doing crossword puzzles, playing board games or cards, participating in organized group discussions, and playing musical instruments, among others. Some of these activities intended as “cognitive” are also social ones, and therefore the interpretation of their role is biased by another important component of a good cognitive performance, that is, socialization.

The effects of computer training and internet use have also been recently assessed in a randomized controlled trial on 240 healthy elderly participants undergoing either no training or three 4-hour training sessions over 2 weeks, in which however no cognitive measures were taken into account and no positive or negative influence on well-being measures was shown [55]. As in the case of physical activity, the self-reported nature and personal interpretation of leisure type as well as the short time of referral hinder the full interpretation of the results.

Once again, similarly to the preventive aspects of physical exercise, there are still a number of reasons that should motivate physicians to encourage continuous cognitive stimulation in healthy and cognitively impaired subjects. These are based upon the “use it or loose it” concept and include their role as a part of a healthy lifestyle. The latter include the performance of cognitively stimulating activities according to personal interests, abilities and education, and, in demented patients, the prescription of activities that reduce passive behaviors and increase engagement to cognitive and physical activities [56].

### 3.3. Diet

There is strong epidemiologic evidence that, together with physical inactivity, a poor diet is one of the leading causes of death for Americans [57]. Diet therefore plays a crucial role in prevention of age-related chronic disease. Bioactive compounds like antioxidants, mostly contained in fruits and vegetables, are important for the protection against oxidative and nitrosative stress. These processes have been associated with aging itself and the pathophysiology of cognitive impairment and dementia [58].

Decreased food intakes, eating behavior disturbances, and loss of body weight are particularly significant problems among those with AD. A condition of malnutrition has been shown to be associated with a more rapid worsening of AD [59]. However, most of the RCT and prospective cohort studies have focussed on dietary restriction, antioxidants, fish oil, omega-3 fatty acid, and other supplements [60–62] rather than on natural nutrition and diet, or are ongoing [63]. With the exception of one study [44], an association between high dietary antioxidant intake and a decreased risk for AD has been consistently reported [64]. Intervention trials of antioxidant supplementation, however, have demonstrated no major benefit against cognitive impairment [65]. There are several reasons explaining this discrepancy [66]. One key point for it is the largely unexplored relationship between intake of fruits and vegetables, antioxidant micronutrient status, a condition of oxidative stress, and cognitive performance in healthy subjects. There are, however, some hints of biological interactions between these components after evaluation of independent measurements in healthy subjects [67, 68]. It is also likely that when the clinical symptoms of AD appear a large proportion of neuronal cells might already be destroyed and therefore the intervention with antioxidants, especially when a single compound is used instead of a micronutrient network, could come too late.

As far as dietary intervention in dementia prevention is concerned, it is now widely believed that the actions of the antioxidant nutrients alone do not explain the observed health benefits of diets rich in fruits and vegetables, because taken alone, the individual antioxidants and macro/micronutrients studied in clinical trials do not appear to have consistent preventive effects [65]. Fruits and vegetables are thought to represent the best source of antioxidant micronutrients due to synergisms of their components, because they may allow a better bioavailability of protective compounds than single vitamins, and due to their low content in saturated fats. Recently, the effects of dietary counseling on fruit and vegetable intake as well as of improved fruit and vegetable intake on the levels of circulating antioxidants and biomarkers of oxidative stress were studied. One-hundred twenty-nine employees of a University hospital followed a diet consisting of at least five portions of fruits and vegetables per day over three months. Fruit and vegetable intake was monitored, counseling sessions were offered and blood samples were obtained. Several antioxidants were measured over the course of the study along with biomarkers of lipid peroxidation and protein oxidation. A significant increase in several plasma antioxidant micronutrients in the absence of changes of biomarkers of oxidative stress during the course of the study in this health-conscious study population (mostly females, relatively young, well educated) was observed [69]. These results suggest that a nutritional counseling program can lead to improvement in plasma antioxidant status even in a health conscious population of professionals, in which a relevant decrease in biomarkers of oxidative stress is not to be expected. In patients with a typical age-related disease such as AD, showing increased circulating levels of biomarkers of oxidative stress, a targeted nutritional intervention aimed at increasing plasma antioxidant levels and at decreasing the ongoing condition of oxidative stress might prove beneficial in addition to standard therapeutic options.

To follow the natural evolution of dietary and nutrition status among elderly community-dwelling adults with AD, Shatenstein et al. [63] prospectively studied 36 community-dwelling patients in early stages of AD and 58 age-matched cognitively intact community-based controls over a 18-months period. In this study, nutrient intakes from diet and supplements were higher in control subjects, with significant differences in energy, the macronutrients calcium, iron, zinc, vitamin K, vitamin A, and dietary fiber as well as *n*-3 and *n*-6 fatty acids [63]. The authors suggested that suboptimal diet is early in the onset of the disease and that AD patients would benefit from systematic dietary assessment and intervention to prevent further deterioration in food consumption and increased nutritional risk.

In a prospective cohort study of over 3,700 older participants of the Chicago Health and Age Project, high vegetable consumption was associated with a slower rate of cognitive decline over six years after adjusting for age, gender, race, education, cardiovascular-related conditions and risk factors [45]. In this study, the consumption of green leafy vegetables, rich in antioxidant micronutrients like carotenoids, showed the strongest inverse linear association with the rate of cognitive decline. The specific protection shown by vegetables and particularly by the green leafy ones appears to be in disagreement with the concept that fruit and vegetable consumption might be beneficial in the frame of a generally healthy lifestyle, as health-conscious individuals tend to consume both fruits and vegetables.

Healthy diet in general and the Mediterranean regimen in particular have been recently shown to affect risk for and mortality from AD [43, 45–47, 70]. The existing evidence does not support the recommendation of specific supplements, foods, or diets for the prevention of AD [44]. However, a review of 34 studies in the areas of dietary restriction, antioxidants and Mediterranean diet provided evidence that nutritional interventions against dementia and AD have a great potential of influencing dementia development [61].

## 4. Conclusions and Practical Recommendations

There is increasing recognition for the role of lifestyle in the adequate approach to risk assessment and management of dementia. Evaluation and counselling by the physician is likely to strongly influence awareness of dementia from patients and caregivers and hopefully disease progression. From a pragmatic point of view, there are several lifestyle aspects of the patient about which dementia care professionals should be informed in detail.

Although consensus is rare in the dementia research community, there is widespread agreement that the development of dementia prevention strategies is of paramount importance. If effective prevention strategies could be identified and standardized, the biomarker [71] as well as the cognitive [19] and general [72] assessment of preclinical dementia pathology might be included among other screening tools in the field of preventive medicine.

Due to the rapid increase of dementia burden, lifestyle factors described above as well as their amelioration should already be part of decision making while awaiting for results of large prospective cohort studies. Insufficient evidence to make a firm recommendation against dementia development is due to the lack of coordination of preventive strategies and should not be used as a reason to disregard key components of a healthy behaviour enabling both elderly subjects to remain cognitively fit or patients with dementia to slow disease progression. Dementia is not a destiny, and by influencing lifestyle a likely significant decrease of the number of patients (prevalence) as well as delay of the disease manifestation (incidence) will be achieved.

## Figures and Tables

**Table 1:** RCT and prospective cohort studies on the effects of physical activity, cognitive and social activity, and natural nutrition on cognition and or dementia risk in healthy or demented patients aged 70 years and older.

	Diagnosis	Design	Number	Outcome	Intervention/Measure	Results
PHYSICAL ACTIVITY						

Baum et al. [24]	Mild Dementia (mean MMSE 21/30)	RCT	20	Cognition	Strength training or recreational therapy 6 months	Improved MMSE with physical activity
Van de Winkel et al. [25]	Severe Dementia (mean MMSE 13/30)	RCT	15	Cognition	Physical activity + music or conversation 3 months	Improved MMSE with physical activity
Weuve et al. [33]	Healthy women	Prospective cohort Nerses' Health Study	766	Cognition	Physical activity and walking	Better cognitive function/less cognitive decline with physical activity and walking
Stevens and Killeen [26]	Mild and Severe Dementia (MMSE 9-23/30)	RCT	75	Clock drawing test	Physical activity or social visit or none	Slower cognitive decline with physical activity
Lautenschlager et al. [27]	Subjective memory impairment	RCT	308	Dementia	Education and usual care versus physical activity for 6 mos	(Modest) Cognitive improvement at 18 mos
Brown et al. [28]	Healthy subjects	RCT	134	Cognition	Balance versus general training	Cognitive improvement at 6 months
Verghese et al. [29]	Healthy subjects	Prospective Cohort	469	Dementia	Physical activity versus Leisure/Cognitive Activity	Decreased risk for dementia
Abbott et al. [30]	Healthy subjects	Prospective cohort Honolulu Asia Aging	2257	Dementia	Physical activity, walking	Decreased risk for dementia
Larson et al. [31]	Healthy subjects	Prospective cohort	1740	Dementia	Physical exercise	Decreased risk for dementia
Cassilhas et al. [32]	Healthy subjects	RCT	62	Cognition	Moderate or High-level resistance training	Improvement of cognition with both levels of resistance training

SOCIAL, COGNITIVE AND LEISURE ACTIVITY						

Scarmeas et al. [36]	Healthy subjects	Prospective cohort	1772	Dementia	Leisure activities	Decreased risk for dementia
Wang et al. [37]	Healthy subjects Kungsholmen project	Prospective cohort	152	Dementia	Intellectual and social stimulation	Decreased risk for dementia
Verghese et al. [29]	Healthy subjects	Prospective cohort	469	Dementia	Leisure activities	Decreased risk for dementia
Karp et al. [38]	Healthy subjects Kungsholmen project	Prospective cohort	776	Dementia	Mental, physical or social activity versus two or more	Decreased risk for dementia with increasing number of activities
Verghese et al. [35]	Healthy subjects of the Bronx Aging Study	Prospective cohort	437	Amnestic MCI	Leisure activities	Decreased risk for MCI with increasing number of activities
Willis et al. [39]	Healthy subjects	RCT	2832	Cognition	Verbal episodic memory training versus Inductive reasoning training versus visual search and identification training versus no training	Improved cognition with any training type
Helzner et al. [40]	AD	Prospective cohort	287	Cognition	Leisure activities	No association
Wilson et al. [41]	Healthy subjects from Rush Memory and Aging Project	Prospective cohort	770	MCI	Cognitive activities	Decreased risk for MCI with increased cognitive activity
Karp et al. [42]	Healthy subjects Kungsholmen project	Prospective cohort	931	Dementia	Work complexity	Decreased risk for dementia with increasing work complexity
	Healthy subjects Kungsholmen project	Prospective cohort	506	Dementia	Neuroticism and extraversion	Decreased risk for dementia with low neuroticism and high extraversion

NUTRITION						

Barberger-Gateau et al. [43]	Healthy subjects	Prospective Cohort	8085	Dementia	Fruit and vegetable intake versus fish and omega-3 fat	Decreased risk for dementia with high fruit, vegetable, fish and omega-3 fat intake
Luchsinger et al. [44]	Healthy subjects	Prospective Cohort	980	AD	Daily intake of calories, carbohydrates, fats and proteins	Increased risk for AD with increased caloric and fat intake
Morris et al. [45]	Healthy subjects	Prospective Cohort	1718	Cognition	High versus low fruit and vegetable intake	Slower cognitive decline with high vegetable intake
Scarmeas et al. [46, 47]	Healthy subjects	Prospective Cohort	2258	AD	Adherence to Mediterranean diet versus no adherence	Decreased risk for AD with increased adherence to Mediterranean diet
Morris et al. [45]	Healthy subjects	Prospective Cohort	1041	AD	Nutritional folate, B12, B6 vitamins	No association
